# Phenotypic homogeneity in childhood epilepsies evolves in gene-specific patterns across 3251 patient-years of clinical data

**DOI:** 10.1038/s41431-021-00908-8

**Published:** 2021-05-24

**Authors:** David Lewis-Smith, Shiva Ganesan, Peter D. Galer, Katherine L. Helbig, Sarah E. McKeown, Margaret O’Brien, Pouya Khankhanian, Michael C. Kaufman, Alexander K. Gonzalez, Alex S. Felmeister, Roland Krause, Colin A. Ellis, Ingo Helbig

**Affiliations:** 1 Translational and Clinical Research Institute, Newcastle University, Newcastle-upon-Tyne, UK; 2 Department of Clinical Neurosciences, Royal Victoria Infirmary, Newcastle-upon-Tyne, UK; 3 The Epilepsy NeuroGenetics Initiative (ENGIN), Children’s Hospital of Philadelphia, Philadelphia, PA USA; 4 Department of Biomedical and Health Informatics (DBHi), Children’s Hospital of Philadelphia, Philadelphia, PA USA; 5 Division of Neurology, Children’s Hospital of Philadelphia, Philadelphia, PA USA; 6 Department of Neurology, University of Pennsylvania, Perelman School of Medicine, Philadelphia, PA USA; 7 Luxembourg Centre for Systems Biomedicine, University of Luxembourg, Esch-sur-Alzette, Luxembourg

**Keywords:** Genetics research, Prognostic markers, Genetic testing, Diagnostic markers

## Abstract

While genetic studies of epilepsies can be performed in thousands of individuals, phenotyping remains a manual, non-scalable task. A particular challenge is capturing the evolution of complex phenotypes with age. Here, we present a novel approach, applying phenotypic similarity analysis to a total of 3251 patient-years of longitudinal electronic medical record data from a previously reported cohort of 658 individuals with genetic epilepsies. After mapping clinical data to the Human Phenotype Ontology, we determined the phenotypic similarity of individuals sharing each genetic etiology within each 3-month age interval from birth up to a maximum age of 25 years. 140 of 600 (23%) of all 27 genes and 3-month age intervals with sufficient data for calculation of phenotypic similarity were significantly higher than expect by chance. 11 of 27 genetic etiologies had significant overall phenotypic similarity trajectories. These do not simply reflect strong statistical associations with single phenotypic features but appear to emerge from complex clinical constellations of features that may not be strongly associated individually. As an attempt to reconstruct the cognitive framework of syndrome recognition in clinical practice, longitudinal phenotypic similarity analysis extends the traditional phenotyping approach by utilizing data from electronic medical records at a scale that is far beyond the capabilities of manual phenotyping. Delineation of how the phenotypic homogeneity of genetic epilepsies varies with age could improve the phenotypic classification of these disorders, the accuracy of prognostic counseling, and by providing historical control data, the design and interpretation of precision clinical trials in rare diseases.

## Introduction

Over the last decade, genetic discovery in the epilepsies has been enabled by the ability to analyze genomic data in thousands of individuals [1, 2]. A major bottleneck in translating genetic findings into clinical actions is the limited ability to interpret the relevance of genetic features to the varied phenotypes encountered in clinic. In contrast to genomic data, analysis of detailed phenotypic information remains a largely manual, non-scalable task. Consequently, the resolution of large gene discovery studies is limited to broad epilepsy types or syndromes rather than specific phenotypic details [1, 2]. Conversely, the largest detailed phenotypic studies include only a few hundred individuals, typically focusing on a single etiology without harmonized comparison with the phenotypic repertoire of genetic epilepsies as a whole [3, 4].

Clinical phenotype data are increasingly available in electronic medical records (EMR), which have become the standard form of medical record [5]. However, the complexity of clinical data makes harmonization critical for computational analysis.

We have previously used phenotypic similarity analysis to demonstrate that specific known genetic etiologies of developmental and epileptic encephalopathies (DEE) are associated with distinctively similar clinical features and to identify *AP2M1* as a novel cause [6, 7]. Phenotypic similarity approaches mimic the human cognitive process of recognizing shared clinical features that give rise to a distinguishable clinical syndrome. Each participant’s features are mapped onto a common framework such as the Human Phenotype Ontology (HPO) for harmonized comparison, weighting rare clinical features more heavily than those that were common in the cohort [8].

However, epilepsies are phenotypically dynamic, currently defined by age-specific syndromes [9]. Assessing phenotypic similarity without reference to age risks overlooking conditions in which age-dependence is a critical component. For example, the phenotypic similarity of *KCNQ2* did not reach statistical significance in a cohort with DEE [7]. It may be that it is the onset of symptoms in the neonatal period rather than the particular clinical features themselves that clinically distinguishes *KCNQ2-*related epilepsies. While the HPO includes ways to encode age-based features within broad categories such as “neonatal onset” or “childhood onset”, a more nuanced approach may be necessary to identify differences such as the later onset of Dravet syndrome when caused by variants in *PCDH19* rather than *SCN1A* [10]. Accordingly, we have analyzed 3251 patient-years of EMR data from 658 patients with 101 distinct genetic etiologies to identify longitudinal footprints of gene-specific associations with single phenotypic features as they evolve with age [11]. While informative, associations with single phenotypic features do not capture the overall gestalt of a syndrome. For example, describing a strong association of *STXBP1* with infantile spasms at 9 months is insufficient to describe the full clinical picture of *STXBP1*-related disorders [3, 11].

Here, we combine phenotypic similarity analysis and longitudinal EMR data mapping to identify the ages at which individuals sharing the same genetic etiology become sufficiently phenotypically homogeneous to be distinguished within a large cohort with similar clinical features [11]. We find that age-related similarity peaks and troughs emerge for specific etiologies, indicating the ages at which the phenotypic constellations of individuals sharing an etiology are particularly homogeneous or heterogeneous. Our approach indicates how large-scale EMR data can be harnessed to uncover the longitudinal phenotypes of genetic disorders, generating evidence for refinement of phenotypic-molecular disease classifications, improved genetic prognostication, and the design and interpretation of clinical trials in rare diseases.

## Materials (subjects) and methods

### Ethics statement

Informed consent for participation was obtained from subjects themselves or, where necessary, their parents. The study was completed per protocol in accordance with the Declaration of Helsinki with local approval by the Children’s Hospital of Philadelphia (CHOP) Institutional Review Board (IRB 15-12226).

### The cohort’s genetic diagnoses, EMR usage, and HPO annotation

**Fig. 1:**
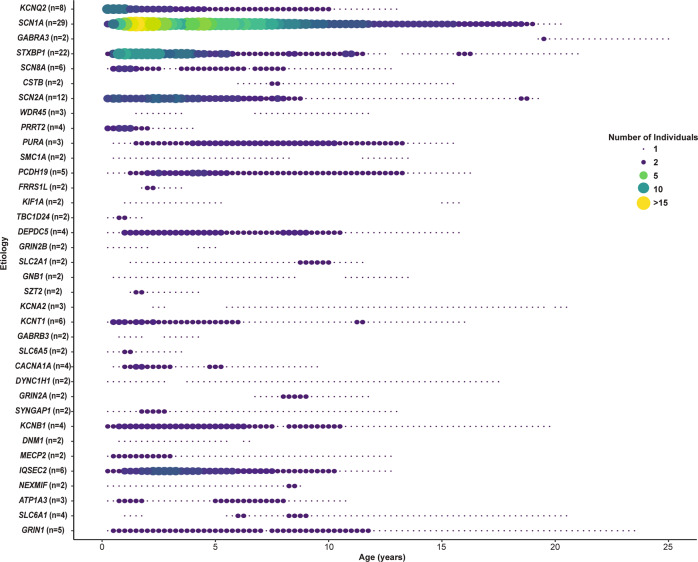
Genetic etiologies of epilepsy demonstrate distinct time-dependent distributions of encounters in the electronic medical records. For each of the 36 genetic etiologies identified in two or more individuals in this cohort, the number of individuals contributing data to each 3-month interval between birth and 25 years is shown. The total number of individuals with a particular genetic etiology is given in brackets after the gene symbol.

Patient recruitment, EMR data extraction, and HPO annotation were performed as described previously [11] and summarized in the supplementary information. In brief, clinical genetic diagnoses and EMR data were collected from 658 individuals recruited from epilepsy and neurogenetic services at Children’s Hospital of Philadelphia. Of 101 genetic etiologies, 36 were identified in multiple individuals (Fig. 1, Supplementary Table S1 and Fig. S2). We refer to the time between an individual’s first and last entries in the EMR as their “EMR usage” and binned this into 3-month intervals for our primary analysis (and on a logarithmic time scale for a supplementary analysis). Only 27 of the 36 etiologies found in multiple individuals were amenable to meaningful age-specific phenotypic similarity analysis because this requires a minimum of two individuals sharing the etiology to have overlapping EMR usage. We extracted Intelligent Medical Object (IMO) terms based on neurology related ICD10 codes from the EMR. We used the Clinical Text Analysis and Knowledge Extraction System (cTAKES) natural language processing algorithm [12] and manual mapping to create a dictionary translating IMO terms into HPO terms (HPO release version 1.2; 2017-12-12). We annotated individuals’ HPO terms to each 3-month age interval, assuming that phenotypes present at successive EMR encounters were present between, but not before the first or beyond the most recent encounter. All applicable conceptually broader HPO terms were added to each individual’s set of HPO terms for each particular age interval, exploiting the semantic relationships of the HPO.

### Assessment of semantic similarity between pairs of individuals (sim)

The information content *(IC)* of an HPO term at age interval *t* indicates its discriminatory value based on its frequency *(f)* within the cohort with EMR usage at that age. We defined this as *IC*_*t*_ = −log_2_ *f*_*t*_.

The similarity *(sim)* of individuals *P*_*1*_ and *P*_*2*_ at age range *t* was defined as the sum of the information content of their set of shared HPO terms at that age [7]:

$$sim\left( {P_1,P_2} \right)_t = \mathop {\sum } IC_t\left( {HPO_{P_1t\mathop { \cap }P_2t}} \right)$$

### Determining the relative age-specific phenotypic similarity of a genetic etiology

We created the “PhenSim score”, a measure of an etiology’s overall phenotypic homogeneity compared to age-matched participants. At each age interval *(t), sim* was determined for all possible pairwise combinations of individuals contributing EMR data to that age interval. Next, for each genetic etiology *(x)* ascribed to two or more of these individuals, we identified those pairs in which both individuals had etiology *x* (“gene-positive pairs”). We counted number of gene-positive individuals *(n*_*x*_*)* and calculated the median *sim* score of gene-positive pairs. Then we calculated the median *sim* of each of 100,000 permutated groups *(perm)* of *n*_*x*_ individuals with EMR usage at this age. At each age *t*, we defined the PhenSim score of *x* as the negative logarithm of the rank of its median *sim* within this null distribution with conservative handling of ties:

$$PhenSim_t(x) \!=\! - \log _{10}\frac{{\left( {count\left( {median\;sim_t(perm) \ge median\;sim_t(x)} \right)} \right) \!+\! 1}}{{number\,of\,perm + 1}}$$

Greater PhenSim scores represent greater homogeneity of an etiology relative to individuals under follow-up at the same age. PhenSim scores are cross-sectional, agnostic to individuals’ documented phenotypes prior to their most recent clinical encounter. PhenSim allows for variation in the number of individuals sharing an etiology with EMR usage at different ages, making PhenSim scores comparable across etiologies and ages. Consequently, the longitudinal trajectory of PhenSim of an etiology indicates how its relative phenotypic homogeneity evolves with age. We calculated each etiology’s cumulative PhenSim score as the sum of its PhenSim score across all ages.

### Interpretation of the significance of PhenSim scores

In order to interpret the significance of age- and gene-specific PhenSim scores, we permutated the etiological labels of all 658 participants 1,000,000 times for each unique value of *N*_*x*_, where *N*_*x*_ is the total number of individuals with etiology *x* in the cohort. We calculated the PhenSim score of each of these permutated groups at all age intervals, as above for the true etiologies, generating empirical null distributions of PhenSim for each *N*_*x*_ at each age *t* and cumulative PhenSim scores across all ages.

The raw *p* value of the PhenSim score of etiology *x* at age *t* is given by its conservative rank among those of the permutated groups of size *N*_*x*_:

$$raw\,p - value_{PhenSim_t(x)} = \frac{{\left( {count\left( {PhenSim_t(perm) \ge PhenSim_t\left( x \right)} \right)} \right) + 1}}{{number\,of\,perm + 1}}$$

In addition to the phenotypic features of individuals, this takes account of individuals’ patterns of EMR usage, and the number of individuals with a particular etiology in the cohort. We adjusted *p* values by Holm’s method for the 3600 hypotheses considered (36 etiologies present in multiple individuals and 100 age intervals). For a supplementary *post hoc* analysis we adjusted for only the 600 hypotheses we could test with the data obtained from this cohort.

We assessed the evidence supporting a particularly homogeneous longitudinal phenotype for each etiology using the *p* value of its cumulative PhenSim score (based on its rank among cumulative PhenSim scores obtained from the 1,000,000 permutated groups for each value of *N*_*x*_*)* with Holm’s adjustment for the 36 etiologies.

### Interpreting phenotypic similarity in light of associations with single HPO terms

To investigate whether high phenotypic similarity at a given age coincided with strong associations with single HPO terms we revisited the age-specific associations of each genetic etiology with single terms in this cohort [11]. We focused on etiologies and ages at which we could be 70% confident that we would detect strong associations. The statistical power to detect an association between each gene and a single phenotypic term using Fisher’s exact test was calculated using the *pwr.2p2n.test* function of the *pwr* package within the R Statistical Framework, which was used for all analyses. We selected an odds ratio of 2 as the minimum effect size and a threshold significance level of 0.01 (without adjusting for multiple comparisons because HPO annotations describing the same feature at different levels of precision are highly correlated) to define a strong association between an etiology and a single HPO term of sufficient size and confidence to be potentially useful for distinguishing disorders in clinical practice.

## Results

### Genetic etiologies show unique patterns of EMR usage

EMR usage demonstrated patterns of medical interaction associated with each genetic etiology, often reflecting their known onset and trajectories (Fig. 1 and Supplementary Table S1). EMR usage of *PRRT2* mirrored the infantile onset and resolution of the epilepsy in early childhood [13–15], and of *SCN1A* and *STXBP1* was sustained from infancy through childhood reflecting the chronicity of their related disorders [3, 16–19]. *KCNQ2* and *SCN2A* had both a concentration of EMR usage in the infantile period reflecting self-limited early onset epilepsies and a tail extending through childhood, reflecting DEE [4, 20, 21]. EMR usage was lower in individuals with a molecular diagnosis than those without (Median 2.6 vs 4.3 years, Wilcoxon Rank Sum Test *p* value < 5 × 10^−5^, Supplementary Fig. S2).

### Most genetic etiologies show specific phenotypic homogeneity

**Fig. 2:**
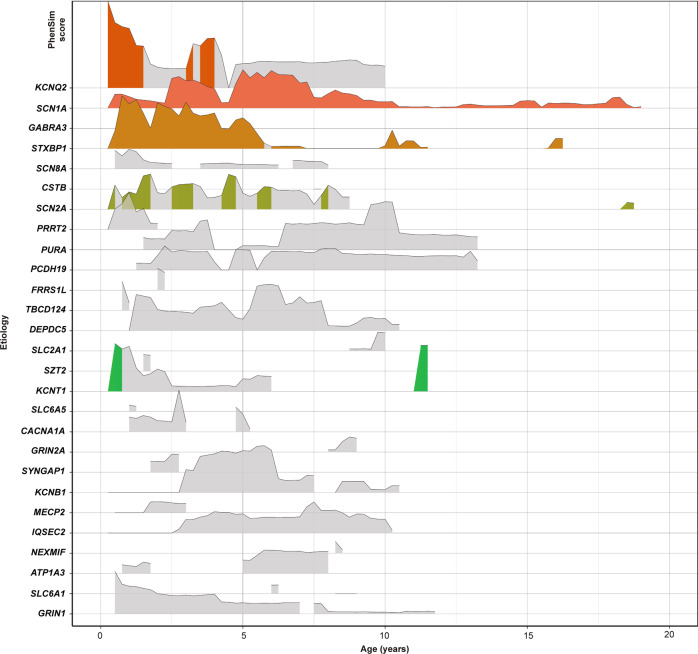
Genetic epilepsies show phenotypic similarities that vary over time. PhenSim scores are shown for the 27 etiologies for which PhenSim could be calculated in this cohort. The height of each ridge indicates PhenSim score at for the corresponding etiology at that age. PhenSim scores significant after Holm’s adjustment for 3,600 hypotheses are shown in color, and nonsignificant PhenSim scores in gray.

11 of 27 etiologies had cumulative PhenSim scores greater than expected by chance (Table 1). The analysis of PhenSim scores over time generated a phenotypic “timescape” for all disease genes found in two or more individuals (Fig. 2 and Supplementary Table S3). Overall, we found significant age-specific PhenSim scores across 140 of 600 (23%) of all 27 genes and age intervals for which phenotypic similarity could be meaningfully analyzed, after Holm’s adjustment for 3600 hypotheses. These spanned five etiologies: *KCNQ2, KCNT1, SCN1A, SCN2A*, and *STXBP1*. All of these had significant PhenSim scores by 6 months of age. A less conservative *post hoc* interpretation based only on the 600 calculable PhenSim scores suggested that 38 further scores could be significant, including that of *GRIN1* at 6 months (Supplementary Table S3 and Fig. S4). While 3-month age bins may be appropriate for infancy when normal development and disease phenotypes evolve rapidly, these may be suboptimal at older ages when there is greater chronological variability in neurodevelopment. Hence, in a supplementary analysis we explored the effect of binning age into 10 intervals of exponentially increasing duration after infancy with the last spanning 15.8–25 years (Supplementary Table S5 and Fig. S6). Compared to fixed 3-month intervals, these wider bins allowed PhenSim scores to be calculated for two additional etiologies *(DNM1* and *KCNA2)* and resulted in greater PhenSim scores (median calculable PhenSim = 0.91 versus 0.70) that were more sustained into adolescence. After Holm’s adjustment, significant PhenSim scores were identified for the same five etiologies and additionally for *GRIN1* and *PRRT2*. The overall proportion of PhenSim scores reaching significance was greater (34/360 = 9.4% versus 140/3600 = 3.9%, odds ratio = 2.58 (95% confidence interval = 1.69–3.84), Fisher’s exact test raw *p* value < 1.1 × 10^−5^).

**Table 1:** The evidence for distinctive trajectories of phenotypic homogeneity and EMR usage.

Genetic etiology	Number of individuals with the etiology	Cumulative PhenSim score from birth to age 25 years	Raw *p* value	Holm’s adjusted *p* value
*KCNQ2*	8	62.1	1.00E−06	3.60E−05
*SCN1A*	29	43.3	1.00E−06	3.60E−05
*SCN2A*	12	33.4	1.00E−06	3.60E−05
*STXBP1*	22	38.8	1.00E−06	3.60E−05
*IQSEC2*	6	26.3	1.05E−04	0.00336
*PCDH19*	5	36.4	1.22E−04	0.00378
*DEPDC5*	4	41.1	1.94E−04	0.00582
*KCNT1*	6	22.2	2.30E−04	0.00667
*PURA*	3	43.5	3.76E−04	0.0105
*GRIN1*	5	26.4	4.63E−04	0.0125
*KCNB1*	4	32.1	6.70E−04	0.0174
*SCN8A*	6	9.89	0.00223	0.0556
*ATP1A3*	3	15.4	0.00883	0.212
*CACNA1A*	4	9.25	0.0273	0.627
*PRRT2*	4	6.64	0.0511	1.00
*SYNGAP1*	2	3.39	0.216	1.00
*MECP2*	2	3.04	0.234	1.00
*SLC2A1*	2	2.22	0.284	1.00
*GRIN2A*	2	2.17	0.288	1.00
*FRRS1L*	2	1.96	0.302	1.00
*TBC1D24*	2	1.82	0.313	1.00
*SZT2*	2	1.60	0.331	1.00
*SLC6A1*	4	0.858	0.360	1.00
*NEXMIF*	2	0.749	0.413	1.00
*SLC6A5*	2	0.577	0.431	1.00
*GABRA3*	2	0.325	0.459	1.00
*CSTB*	2	0.00	1.00	1.00

The empirical *p* value derived from 1,000,000 permutations indicating the probability of observing a cumulative PhenSim score at least as great as that of each etiology due to chance is provided before and after Holm’s adjustment for 36 genetic etiologies.

### EMR usage powers phenotypic similarity analysis

**Fig. 3:**
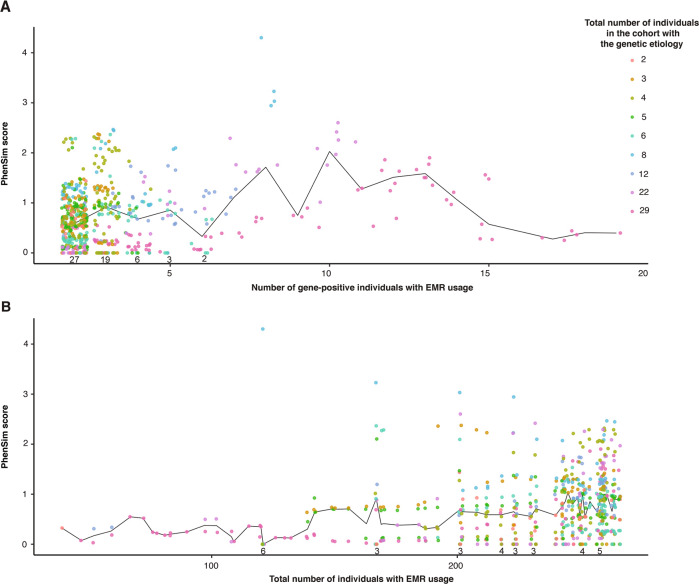
The relationship between PhenSim and the number of individuals with EMR usage at the corresponding age. The effect of the number of (**A**) gene-positive and (**B**) total individuals with EMR usage on PhenSim scores at the corresponding age. The number of etiologies with PhenSim scores of zero at each number of gene-positive individuals where this was observed is shown above the x-axis in (**A**). The median PhenSim score for each value on the x-axes is shown by the black lines.

A minimum of two individuals sharing an etiology and EMR usage at the age of interest is necessary to calculate PhenSim. The median PhenSim score, where calculable, was 0.70. However, PhenSim scores >1.3 (i.e., the etiology is found in the top 5% of possible median *sim* values) were observed only at ages where >120 individuals had overlapping EMR usage (Fig. 3). We explored the relationship between the number of individuals with EMR usage and the corresponding PhenSim scores after permutation of etiological labels to confirm that our results had not arisen as artifacts of this (Supplementary Fig. S7). Even with small numbers of simulated gene-positive individuals (where PhenSim is more sensitive to extreme *sim* values) the probability of each PhenSim score being >0.25 by chance is <5%. PhenSim scores gradually increase with the total number of individuals with EMR usage, but even with 240 individuals, the probability of any particular PhenSim score exceeding 0.5 is <5%.

### Patterns of phenotypic similarity are distinct from single phenotype associations

**Fig. 4:**
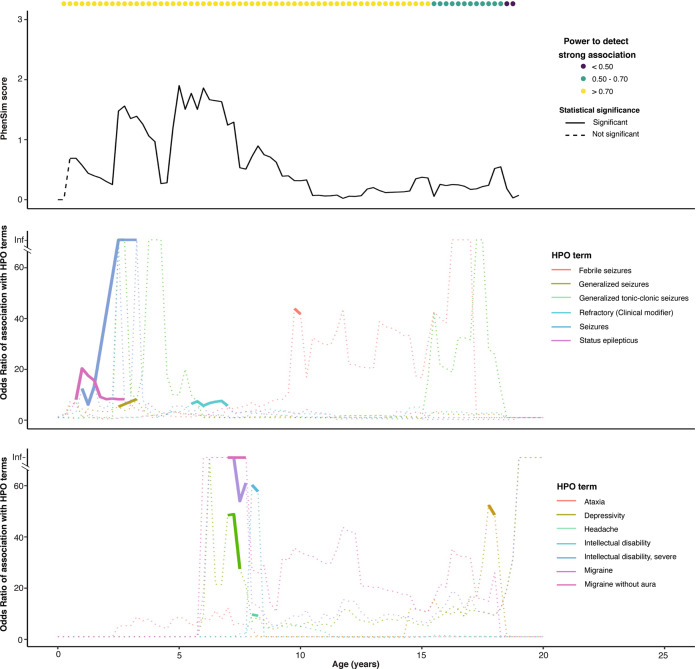
The relationship between strong associations with single phenotypic terms and phenotypic similarity according to age for *SCN1A.* Associations are shown only for terms reaching a strong association at any given age. Associations are plotted as solid lines at ages with a *p*-value < 0.01 and as a dotted line at ages where *p*-value > 0.01. PhenSim scores are shown as solid lines where significant after Holm’s adjustment and dashed lines where not.

In addition to using the 528 HPO terms annotated to this cohort to quantify the overall phenotypic similarity of one individual to another, we have previously tested the association of each etiology to each of these terms at each age [11]. Next we sought to explore the chronological relationship between an etiology’s associations with individual HPO terms and its PhenSim trajectory. We limited our attention to the four most frequent etiologies focusing on ages with at least 70% power to detect strong associations. The chronological relationship between PhenSim and associations with single HPO terms was complex (Fig. 4 and Supplementary Fig. S8). For *SCN1A*, high PhenSim scores occurred at ages with (2.5–2.75 years) and without (4.75–5 years) strong associations with single terms, and all significant associations coincided with non-zero PhenSim scores, although some of these PhenSim scores were lower (at 7.5–7.75 years and 17.75–18 years) than those at ages without strong associations (4.75–5 years, Fig. 4). *KCNQ2* had high PhenSim in early childhood, coinciding with its initial strong association with *Neonatal onset [HP:0003623]* and its ancestor HPO terms (Supplementary Fig. S8A). PhenSim fell briefly then increased, tracking single term associations before remaining approximately 1 from 4.5 to 10 years (after which only a single individual had EMR usage) despite multiple large associations. *SCN2A* (Supplementary Fig. S8B) and *STXBP1* (Supplementary Fig. S8C) had high but variable PhenSim scores fluctuating, at times independently of significant associations with single terms.

## Discussion

Here, we combined two approaches to analyze large-scale phenotype data in known or presumed genetic epilepsies, joining the concepts of longitudinal EMR data mapping with phenotypic similarity analysis. Our results demonstrate that EMR analysis can identify age-specific patterns of clinical encounters and phenotypic homogeneity. These can complement age-specific associations with single phenotypic features to delineate the longitudinal trajectory of genetically defined disorders.

Our approach was able to confirm that some specific genetic epilepsies have age-related clinical features that are sufficiently homogeneous according to real-world EMR data as to make them distinct from a wider cohort. For some, while phenotypic similarity was significant across much of follow-up, the degree of similarity relative to age-matched individuals varied, sometimes falling with age. This may reflect how some gene-defined epilepsies are relatively similar early in the disease but evolve with variable types of seizures and outcomes, for example *SCN1A*-related epilepsies, which include Dravet syndrome and Febrile Seizures plus [17–19, 22]. Our supplementary analysis showed that increased pooling of data made rarer etiologies assessable and increased PhenSim scores in adolescence (where fewer data were available). Overall, this increased the probability of an etiology appearing phenotypically distinct but at the cost of lower chronological precision.

Gene-specific patterns of EMR usage reflected known clinical trajectories. For genes associated with neonatal and infantile epilepsies, including *KCNQ2* [20, 21], *KCNT1* [23], *PRRT2* [15], and *SCN8A* [24], we found that EMR usage was concentrated in the first year of life. This was distributed more evenly across childhood for some DEE genes including *IQSEC2* [25], *SCN1A* [22], *SCN2A* [4], and *STXBP1* [3], but sustained into adolescence for only *SCN1A*. Conversely, we demonstrate the extent to which EMR usage limits EMR-based analyses in these rare diseases: despite a cohort of 658 individuals, only 27 etiologies were amenable to age-specific phenotypic similarity analysis, and at limited ages. This lack of data demonstrates the challenges of investigating the outcomes of genetic epilepsies in adolescence and adulthood [26, 27].

The ages of high phenotypic similarity did not always coincide with those of strong associations with individual phenotypic terms. The trajectory of an etiology’s PhenSim score might depend on age-related variation in the etiology’s strong associations, or where these associations are sustained, variation in the associated term’s information content. We also suspect that a high overall phenotypic similarity may emerge from combinations of phenotypic features that individually are less demonstrably associated with the etiology at the corresponding age. This might be expected in a group of disorders with complex clinical pictures made up of constellations of overlapping sets of many individual phenotypes.

Our approach can delineate the longitudinal history of genetic epilepsies and once a pipeline has been developed, this method is more efficient than manual phenotypic comparison, facilitating harmonization and analysis of large numbers of phenotypic features. From a clinical perspective, age-specific associations and PhenSim trajectories could assist genetically informed prognostic counseling in clinic. Furthermore, phenotypic similarity approaches may benefit diagnosis (for example, providing phenotypic evidence to help interpret variants of uncertain significance), even within months of presentation for a few etiologies, and comparison of a patient’s EMR data to reference cohorts may assist with later (more retrospective) diagnosis more broadly. From a research perspective, this approach could provide quantitative phenotypic evidence for the “lumping” or “splitting” of disorders sharing a genetic etiology according to stratification by variant class, domain, or functional consequences within a single gene, or even multiple genes within a common functional network. Additionally, by delineating the range of clinical trajectories, these studies may generate historical control data for the design and interpretation of precision medicine trials in diseases sufficiently rare to preclude large studies achieving high power through traditional concurrent control arms.

There are several limitations of our study. Our findings are blinkered by the perspective of tertiary care EMR because HPO terms were annotated to the individual’s age at the clinical encounter where they were recorded rather than the ages at which they may have first emerged or persisted to. Firstly, this raises questions about the phenotypic features of individuals we were not able to capture before referral to our service or emerging following their last encounter (after discharge, transfer to another health care provider, or limited by age). Regarding the latter, while it may be reasonable to extrapolate phenotypes that are likely to persist after the last EMR entry such as microcephaly or intellectual disability, this would necessitate assumptions about survival and could not be applied to important dynamic phenotypes such as seizure types. Regarding the former, transient early phenotypes may be missed, and even when recorded, the age at the documentation of a phenotype in the EMR may not correspond to when the phenotype was present (for example, *Neonatal onset [HP:0003623]* in Supplementary Fig. S8A). Secondly, while advances in natural language processing may improve interpretation of the chronology of a recorded phenotype, there is a particular challenge inherent in epilepsy EMR data as seizures are paroxysmal and may continue to be recorded despite coming under control with treatment or having entered natural remission. Consequently, at a routine clinical encounter the patient may not have experienced the documented phenotype for several months or years. Furthermore, we limited our data to neurological features for tractability. Inclusion of non-neurological features might identify novel associations or increase phenotypic distinctiveness.

Secondly, our failure to detect high phenotypic similarity at certain ages could reflect insufficient power rather than the true absence of a recognizable gene-specific syndrome. An age-specific cohort of over 120 individuals was required for PhenSim scores >1.3 to emerge; a number of participants not reached at 41 of 100 age intervals. Additionally, several etiologies including *TSC1*, *TSC2*, *CDKL5*, and *MECP2* were underrepresented because at our center children with the typical syndromes of these are followed in dedicated programs. We hope to include them in future studies.

Thirdly, we grouped individuals according to the gene carrying a diagnostic variant without refining this by protein domain or functional consequences. For example, protein truncating variants in *SCN1A* are more likely than missense variants to cause Dravet syndrome rather than milder epilepsies [28], and the electrophysiological consequences of particular *SCN2A* variants might predict age of onset and treatment response [4]. Stratification by such features might yield greater or more persistent phenotypic similarity for the resulting strata but is likely to require cohorts to be larger and followed up for longer if sufficient EMR usage is to be obtained.

Finally, our data were harmonized using the HPO, which does not currently facilitate the analysis of negated phenotypes (those explicitly documented as absent in the EMR) or drug-specific responses. These could have clinically important distinguishing value, for example the absence of developmental delay, or divergent clinical responses to sodium channel blocking drugs [4, 29].

In summary, we find that phenotypic similarity-based analysis of longitudinal EMR data provides a novel approach to assess etiology-specific patterns of clinical features that aligns with our clinical understanding of age-related epilepsy syndromes. We demonstrate that EMR data can be used to systematically assess not only the association of single phenotypic features but also overall clinical likeness between individuals within discrete age ranges using phenotypic constellations. As the size of cohorts with both genomic and EMR data increases, these frameworks will allow us to analyze the dynamic interplay of phenotypes over time, beyond the capabilities of manual data collection, harmonization, and analysis. Ultimately, they may contribute to improved genetically stratified classification, diagnosis, prognostication, and treatment.

## Supplementary information

Supplementary Material

Table S1

Table S3

Table S5

## Data Availability

Supporting data can be found in the Supporting Information of this article as well as our previous publication [11]. The raw data comprise sensitive patient information and are therefore not openly available. Requests for access should be made to IH.

## Code availability

The code for this study is available at https://github.com/shiva-g/The-Cube.
