# Supplementary material for: Phenotypic homogeneity in childhood epilepsies evolves in gene-specific patterns across 3251 patient-years of clinical data
Source: Eur J Hum Genet. 2021 May 24;29(11):1690–700. doi: 10.1038/s41431-021-00908-8 (PMC8560769; doi:10.1038/s41431-021-00908-8)
Supplement: Supplementary file 1 — Supplementary Material [file 41431_2021_908_MOESM1_ESM.docx]

**Phenotypic homogeneity in childhood epilepsies evolves in gene‑specific patterns across 3,251 patient‑years of clinical data**

**SUPPLEMENTARY INFORMATION**

David Lewis‑Smith^1,2*†^, Shiva Ganesan^3,4,5*^, Peter D. Galer^3,4,5^, Katherine L. Helbig^3,4,5^, Sarah E. McKeown^3,4^, Margaret O’Brien^3,5^, Pouya Khankhanian^6^, Michael C. Kaufman^3,4,5^, Alexander K. Gonzalez^4,5^, Alex S. Felmeister^5^, Roland Krause^7^, Colin A. Ellis^4,5,6^, and Ingo Helbig^3,4,5,6†^

**Affiliations:**

^1^ Translational and Clinical Research Institute, Newcastle University, Newcastle‑upon‑Tyne, UK

^2^ Department of Clinical Neurosciences, Royal Victoria Infirmary, Newcastle‑upon‑Tyne, UK

^3^ Division of Neurology, Children’s Hospital of Philadelphia, Philadelphia, PA, USA

^4^ The Epilepsy NeuroGenetics Initiative (ENGIN), Children's Hospital of Philadelphia, Philadelphia, PA, USA

^5^ Department of Biomedical and Health Informatics (DBHi), Children’s Hospital of Philadelphia, Philadelphia, PA, USA

^6^ Department of Neurology, University of Pennsylvania, Perelman School of Medicine, Philadelphia, PA, USA

^7^ Luxembourg Centre for Systems Biomedicine, University of Luxembourg, Luxembourg

^*^ These authors contributed equally to this work.

^†^ **Corresponding authors:**

David Lewis‑Smith

[david.lewis-smith@newcastle.ac.uk](mailto:david.lewis-smith@newcastle.ac.uk)

<https://orcid.org/0000-0002-1735-8178>

Ingo Helbig

[helbigi@email.chop.edu](mailto:helbigi@email.chop.edu)

<https://orcid.org/0000-0001-8486-0558>

Table of Contents

[Supplementary Methods 3](#_Toc70685257)

[*Annotation of HPO terms* 3](#_Toc70685258)

[*Interpretation of the significance of PhenSim scores* 3](#_Toc70685259)

[*Supplementary analysis of PhenSim using logarithmic age intervals* 4](#_Toc70685260)

[Supplementary Results 5](#_Toc70685261)

[*Description of the cohort* 5](#_Toc70685262)

[**Table S1.** The number of individuals with each genetic etiology and their EMR usage 5](#_Toc70685263)

[**Fig. S2.** The distribution of EMR usage among the cohort 6](#_Toc70685264)

[*PhenSim scores according to linear chronological intervals* 7](#_Toc70685265)

[**Table S3.** The number of individuals with each etiology, and their PhenSim score within each of 100 3‑month age intervals 7](#_Toc70685266)

[**Fig. S4.** PhenSim scores adjusted for the number that could be calculated 7](#_Toc70685267)

[*PhenSim scores according to logarithmic chronological intervals* 8](#_Toc70685268)

[**Table S5.** The number of individuals with each etiology, and their PhenSim scores within each of 10 age intervals of increasing duration 9](#_Toc70685269)

[**Fig. S6.** The effect of increasing chronological bins width with age 9](#_Toc70685270)

[Fig. S7. The relationship between phenotypic similarity and the number of individuals with EMR usage at the corresponding age, for simulated groups 10](#_Toc70685271)

[*The chronological relationship between associations and phenotypic homogeneity* 11](#_Toc70685272)

[**Fig. S8A.** *KCNQ2* 11](#_Toc70685273)

[**Fig. S8B.** *SCN2A* 12](#_Toc70685274)

[**Fig. S8C.** *STXBP1* 13](#_Toc70685275)

[References used in the supplementary information 14](#_Toc70685276)

# **Supplementary Methods**

## *Annotation of HPO terms*

We obtained EMR data from the Clarity database (EPIC, 1979 Milky Way, Verona, WI 53593). We extracted Intelligent Medical Object problems and diagnosis terms (IMO, Intelligent Medical Objects, Inc. 60 Revere Drive, Suite 400, Northbrook, IL 60062) based on a selection of neurology‑related ICD10 codes (F00‑F99, G00‑G99, P90, Q00‑Q07, R25‑R29, R40‑R49, R56, R62, R90, and R94.01) documented within the EMR by healthcare providers at each encounter. We used the Clinical Text Analysis and Knowledge Extraction System (cTAKES) natural language processing algorithm (1) to create a preliminary dictionary that translated these IMO terms into HPO terms (HPO release version 1.2; data version releases/2017–12–12; downloaded on 10 March 2018, corresponding to the time of our initial analyses). We manually reviewed the dictionary prior to use, mapping IMO terms related to diseases and epilepsy syndromes to conceptually broad HPO terms to avoid false positive annotation, as even the typical features of syndromes may not be present in all individuals at all ages at which they carry the diagnosis.

Then we annotated individuals with applicable HPO terms for each of 100 discrete 3-month intervals between birth and age 25 years, assuming that phenotypes present at successive EMR encounters were present between these, but not beyond the most recent encounter. All applicable conceptually broader HPO terms (hypernyms) were added to each individual’s set of HPO terms for each particular age interval, using a method that we refer to as “propagation” that exploits the relationships between phenotypic concepts within the HPO (2, 3). Dictionary mapping of neurological EMR data generated 528 unique HPO terms to describe this cohort. Binning of HPO annotations into 3‑month intervals from birth to 25 years yielded a median of 142 individuals (range 5–266) contributing to each time period and a total of 286,085 HPO term annotations (3).

## *Interpretation of the significance of PhenSim scores*

In the primary analysis (results shown in Fig. 2), we adjusted p‑values conservatively by Holm’s method for all 36 etiologies and 100 age intervals (3,600 hypotheses) at which we set out to calculate PhenSim where possible. However, because of the limits of EMR usage (for a PhenSim score to be calculated for an etiology requires at least two individuals with the etiology to have EMR usage at the correspond age), 3,000 of these PhenSim scores could not be calculated (PhenSim = “NA”). The ages at which multiple individuals sharing an etiology are found to share EMR usage (and have any calculable PhenSim score) is phenotypically informative in itself (these indicate when the disorder is sufficiently clinically manifest to require medical evaluation), even if the unitary phenotypic features of those individuals are are not similar enough to produce a PhenSim score above 0. Consistent with this and the adjustment for 3,600 hypotheses, we included PhenSim scores of “NA” in the empirical null distribution used to calculate the raw p‑values of PhenSim in the primary analysis, treating them as being lower than 0. Thus, the null distribution of PhenSim scores for each age an etiology included 1,000,000 values (all of the permutated groups) and it was theoretically possible (but not observed) for a.PhenSim score of 0 to be significant. A significant PhenSim score of 0 would indicate that the etiology was found to make individuals more likely to have overlapping EMR usage at the corresponding age than expected by chance, even if their individual clinical features captured by the HPO were not informatively similar.

We also conducted a less conservative supplementary analysis of significance by performing Holm’s adjustment according to the 600 PhenSim scores that could be calculated from real data ([Fig. S4](#_Figure_S4_PhenSim)). For consistency, we removed PhenSim scores of “NA” from the empirical null distribution used to calculate the raw p‑value of each PhenSim score. Thus, for this supplementary analysis, the null distribution of PhenSim scores for each age and etiology often included fewer than 1,000,000 values. This resulted in higher raw p‑values, but with subsequent adjustment for fewer hypotheses.

## *Supplementary analysis of PhenSim using logarithmic age intervals*

We undertook a supplementary analysis to explore the effects of increasing the breadth of age bins with advancing age. This attempts to account for the clinical observation that the rate of neurodevelopment slows with age and that early onset epilepsy syndromes (such as Ohtahara syndrome) tend to present within a very narrow age range compared to juvenile onset syndromes (such as juvenile myoclonic epilepsy). We defined 10 bins with 7 on a logarithmic scale after infancy, which was divided into three bins. The final bin spanned 15.8–25 years. Raw p‑values were calculated from empirically derived distributions of PhenSim scores using 1,000,000 permutations, and adjusted by Holm’s method for 360 hypotheses (36 etiologies and 10 age intervals, see [PhenSim scores according to logarithmic chronological intervals](#_PhenSim_scores_according)).

# **Supplementary Results**

## *Description of the cohort*

Patient recruitment, EMR data extraction, and HPO annotation were performed as summarized above and described previously (3). At the time of analysis, the cohort contained information from 658 individuals with established epilepsy of presumed genetic etiology or neurogenetic diagnoses typically associated with epilepsy recruited from neurology outpatient and inpatient settings at Children’s Hospital of Philadelphia. Their epilepsies included DEE (n = 268), focal epilepsies (n = 156), and genetic generalized epilepsies (n = 97). Of 101 causative genetic etiologies, 36 were identified in two or more individuals (Fig 1 and Table S1). Diagnoses were reached by both clinical and research pathways including gene panel sequencing (n = 100), exome sequencing (n = 109), single‑nucleotide polymorphism arrays (n = 9), and single‑gene tests (n = 14).

### **Table S1.** The number of individuals with each genetic etiology and their EMR usage

*See accompanying file*


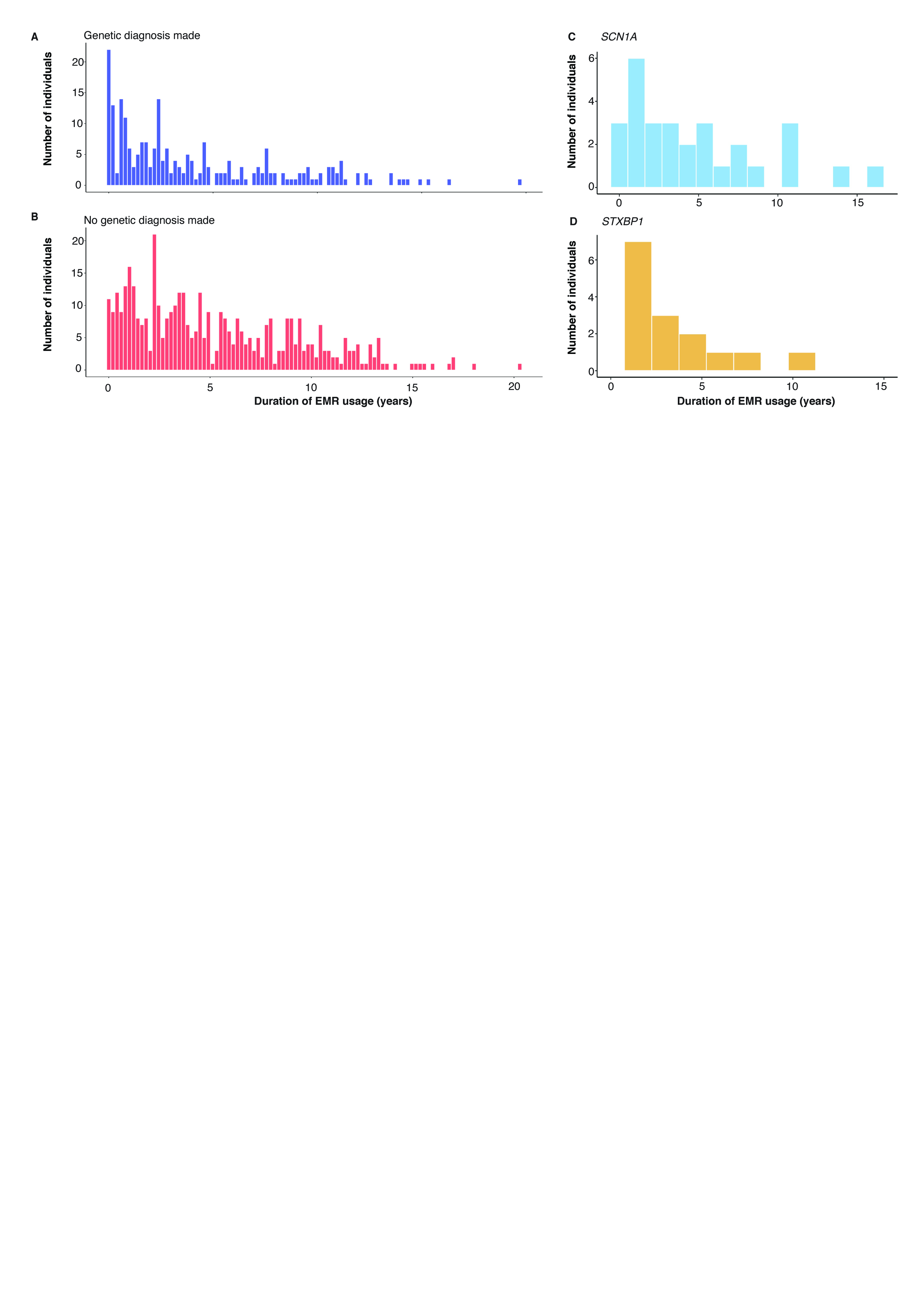


### **Fig. S2.** The distribution of EMR usage among the cohort

The entire cohort, stratified by **(A)** the presence or **(B)** absence of a genetic diagnosis, and for the most common etiologies: **(C)** 29 individuals with a diagnostic *SCN1A* variant, and **(D)** 22 individuals with a diagnostic *STXBP1* variant. The duration of EMR usage for those without a genetic diagnosis was higher than for those with (Median 4.36 vs 2.61 years, Wilcoxon Rank Sum Test with continuity correction W = 39,931, raw p‑value = 4.68 x 10^-5^). The duration of EMR usage for those with an *SCN1A* disorder was higher than for those with an *STXBP1* disorder (Median 3.70 vs 1.64 years, Wilcoxon Rank Sum Test with continuity correction W = 446, raw p‑value = 0.0161). The former may reflect the greater number of investigations, monitoring, or the complexity of health care for those with unexplained disorders when compared to those with a known etiology. The latter reflects practice at our center, which is an international referral center for *STXBP1* disorders and consequently, individuals in this cohort with *STXBP1* disorders may be more likely to have been seen only once or a few times to provide expert opinions while remaining under the care of their local health care network.

## *PhenSim scores according to linear chronological intervals*

### **Table S3.** The number of individuals with each etiology, and their PhenSim score within each of 100 3‑month age intervals

*See accompanying file*





### **Fig. S4.** PhenSim scores adjusted for the number that could be calculated

PhenSim scores are shown for the 27 etiologies for which PhenSim could be calculated in this cohort. The height of each ridge indicates the PhenSim score of the corresponding etiology at that age and is identical to Fig. 2. However, in Fig. 2, p‑values were adjusted by Holm’s method for all 36 etiologies and 100 age intervals (3,600 hypotheses) for which we set out to calculate PhenSim where possible. In this analogous figure, p‑values were adjusted by Holm’s method for only those 27 genes at the ages where this could be calculated (600 hypotheses) after removing the corresponding “NA” values from the empirical null distribution used to calculate the raw p‑values of PhenSim (See [Interpretation of the significance of PhenSim](#_Interpretation_of_the)). As for Fig. 2, significant PhenSim scores are shown in color, and nonsignificant PhenSim scores in gray. This resulted in 178 of 600 PhenSim scores (30%) being deemed significant, 38 more than in the primary analysis. No significant PhenSim scores from the primary analysis became nonsignificant. There was a wider range of ages at which PhenSim became significant for *KCNQ2, KCNT1,* and *SCN2A,* and *GRIN1* became significant between 3­6 months of age.

## *PhenSim scores according to logarithmic chronological intervals*

This supplementary analysis resulted in PhenSim scores for 29 of 36 etiologies present in multiple individuals, with the addition of two more to those from in the primary analysis: *DNM1* and *KCNA2* (Table S5 and Fig. S6)*.* Compared to the primary analysis using fixed 3-month age intervals, this increased the proportion of etiologies and time intervals for which PhenSim could be calculated: 126/360 (35%) versus 600/3,600 (17%), odds ratio = 2.69 (95% confidence interval = 2.11–3.42), Fisher’s exact test raw p‑value < 2x10^‑15^.

A smoothing effect was seen with increased bin width resulting in PhenSim scores that varied less with age, with less of the fall in PhenSim seen with increasing age during the primary analysis. Additionally, the significance of PhenSim scores was more sustained for *KCNQ2* and *SCN2A* than in the primary analysis*.* The calculable PhenSim scores tended to be greater than those found in the primary analysis (median 0.905 vs 0.705, two-sided Wilcoxon Rank Sum Test with continuity correction W = 44,340, raw p‑value < 0.003). Thus, extending age intervals to follow the rate of neurodevelopment more closely than rigid 3-month intervals may help to detect phenotypic similarity by making the analysis less sensitive to small amounts of chronological variability in the precise age at which particular phenotypes manifest between individuals.

The proportion of hypothesized PhenSim scores reaching p‑values < 0.05 after Holm’s adjustment was greater than in the primary analysis: 34/360 (9.4%) versus 140/3,600 (3.9%), odds ratio = 2.57 (95% confidence interval = 1.69‑3.84), Fisher’s exact test raw p‑value < 2 x 10^-5^. *GRIN1* and *PRRT2* reached significance for single age intervals, in addition to the five most common etiologies that were significant in the primary analysis. Hence, greater capture of individuals with EMR usage and a greater number of HPO annotations per interval compared to the narrower intervals of the primary analysis may increase the sensitivity of the analysis for rarer forms of genetic epilepsy, albeit at the price of chronological precision.

### **Table S5.** The number of individuals with each etiology, and their PhenSim scores within each of 10 age intervals of increasing duration

*See accompanying file*





**Fig. S6.** The effect of increasing chronological bins width with age

Age is shown on the x‑axis on a logarithmic scale with the limits of the 10 age intervals indicated in italics and dashed vertical lines. As for Fig. 2 and Fig. S4, significant PhenSim scores are shown in color and nonsignificant PhenSim scores in gray.

## **Fig. S7.** The relationship between phenotypic similarity and the number of individuals with EMR usage at the corresponding age, for simulated groups





We randomly generated 100 simulated diagnostic groups for each of 9 unique values of *N_x_* (the number of individuals in the cohort with a particular true etiology, *x),* giving a total of 900 simulated etiological groups. Analogously to Fig. 3, for each of these values of *N_x_,* we show the effect of the number of **(A)** simulated gene‑positive and (**B)** total individuals with EMR usage on the PhenSim score of the simulated etiological group at the corresponding age. In **(A)** the number of simulated etiological groups with PhenSim equal to 0 for each number of simulated gene‑positive individuals with EMR usage is given below the x‑axis. In both panels the 95‑percentile of PhenSim score for each number of individuals with EMR usage is shown as a black line. Note that unlike the median in Fig. 3, this 95‑percentile of PhenSim is usually 0 and always below 0.4 in both panels.

## *The chronological relationship between associations and phenotypic homogeneity*





### **Fig. S8A.** *KCNQ2*

Only terms sufficiently associated with the etiology to reach an odds ratio > 2 and raw p‑value < 0.01 at some age are shown. Associations are plotted as solid lines at ages with a p‑value < 0.01 and as a dotted line at ages where p‑value > 0.01, and PhenSim scores as solid lines where significant after Holm’s adjustment for 3,600 hypotheses and dashed lines where not. The power to detect an association with an HPO term with odds ratio > 2 and raw p‑value < 0.01 is shown at the top of each panel.





### **Fig. S8B.** *SCN2A*





### **Fig. S8C.** *STXBP1*

## **References used in the supplementary information**

1. Savova GK, Masanz JJ, Ogren PV, Zheng J, Sohn S, Kipper-Schuler KC, et al. Mayo clinical Text Analysis and Knowledge Extraction System (cTAKES): architecture, component evaluation and applications. J Am Med Inform Assoc. 2010;17(5):507-13.

2. Galer PD, Ganesan S, Lewis-Smith D, McKeown SE, Pendziwiat M, Helbig KL, et al. Semantic Similarity Analysis Reveals Robust Gene-Disease Relationships in Developmental and Epileptic Encephalopathies. Am J Hum Genet. 2020;107(4):683-97.

3. Ganesan S, Galer PD, Helbig KL, McKeown SE, O’Brien M, Gonzalez AK, et al. A longitudinal footprint of genetic epilepsies using automated electronic medical record interpretation. Genet Med. 2020;22:2060-70.
